# CD1a Expression in Oral Dysplastic Lesions: An Immunohistochemical Analysis 

**DOI:** 10.30476/dentjods.2025.106737.2685

**Published:** 2026-09-01

**Authors:** Parsa Eftekhari Moghadam, Saede Atarbashi-Moghadam

**Affiliations:** 1 Undergraduate Student, Research Committee, School of Dentistry, Shahid Beheshti University of Medical Sciences, Tehran, Iran.; 2 Dept. of Oral and Maxillofacial Pathology, School of Dentistry, Shahid Beheshti University of Medical Sciences, Tehran, Iran.

**Keywords:** Leukoplakia, Dysplasia, Langerhans cells, CD1a

## Abstract

**Background:**

Leukoplakia is among the most prevalent oral potentially malignant disorders, and its association with oral epithelial dysplasia (OED) markedly increases the risk of malignant transformation. Although histopathological grading systems have evolved, accurately predicting which lesions will undergo malignant progression remains a significant challenge. CD1a, a marker expressed by Langerhans cells, may reflect local immune responses and serve as a potential indicator of dysplastic progression.

**Purpose:**

Given the inconsistencies in previous findings, the present study aimed to assess CD1a-positive cells in OED associated with leukoplakia and investigate their potential role in the pathogenesis of these lesions.

**Materials and Method:**

In this retrospective cross-sectional study, 60 formalin-fixed paraffin- embedded tissue samples were analyzed, comprising 53 OED cases and 7 control samples. Immunohistochemical staining was performed using a monoclonal anti-CD1a antibody. CD1a-positive cells were counted in five high-power fields (40×) in hotspot regions. The severity of dysplasia was categorized according to both 2022 WHO classification and binary grading system; inflammation severity was also scored. Statistical analyses were conducted using the Shapiro-Wilk test, Spearman’s correlation, the Mann-Whitney U test, and the Kruskal-Wallis test in SPSS version 28.

**Results:**

CD1a expression was elevated in dysplastic tissues compared to controls; however, this difference was not statistically significant (*p*= 0.071). According to the binary grading system, CD1a levels were significantly higher in high-risk lesions than in low-risk ones (*p*= 0.044). Although the correlation between CD1a expression and inflammation severity was not statistically significant (*p*= 0.137), the association between severity of inflammation and the grade of dysplasia was statistically significant (*p*= 0.015 for WHO classification; *p*= 0.023 for binary grading). No statistically significant association was found between CD1a expression and patient age, gender, or lesion location.

**Conclusion:**

CD1a may contribute to the process of dysplastic transformation and serve as an indicator of local immune activation. Its increased expression in higher-grade dysplasia was associated with more pronounced inflammatory cell infiltration and a more immune-active microenvironment.

## Introduction

Oral leukoplakia is among the most prevalent oral potentially malignant disorders (OPMDs), clinically defined as white plaques or patches of the oral mucosa that cannot be scraped off. Its rate of malignant transformation is variable, ranging from 1.1% to over 40.8%. A key histopathological criterion used to assess the malignant potential of leukoplakia is oral epithelial dysplasia (OED), which is characterized by cellular atypia and disruption of normal oral epithelial maturation and stratification [ 1 ]. The presence of OED in leukoplakia is correlated with an increased risk of progression to carcinoma. As the severity of OED increases, there is a higher chance of malignant transformation [ 2 ].

WHO proposed a three-tier grading system (mild, moderate, and severe) to classify OED, most recently updated in 2022 [ 3 ]. In addition, a binary grading system (low-risk and high-risk) was also introduced to improve interobserver consistency and clinical decision-making. Recent findings suggest that this approach, which is based on WHO microscopic criteria, may provide a more practical and consistent tool, particularly among clinicians and pathologists with different levels of experience [ 4 ].

Langerhans cells, a subset of dendritic cells, play a key role in immunosurveillance and immune response regulation. These cells recognize, process, and present antigens to T lymphocytes, ultimately generating tumor-specific effector T cells that can inhibit malignant progression [ 5 - 6 ]. Immature Langerhans cells, identifiable by CD1a expression, have a high capacity for antigen uptake and are crucial for maintaining immune surveillance in mucosal and cutaneous tissues [ 7 ]. 

The immunosurveillance and immunoediting hypotheses describe the interaction between the host and malignant cells in three phases including elimination, equilibrium, and escape. When immunosurveillance fails, cancer cells use cytokines and other factors to promote growth and invasion [ 8 ]. During the early stages of dysplasia or malignant transformation, Langerhans cells attempt to eliminate damaged cells [ 6 ].

Given that histological morphology alone may be insufficient to reliably predict the malignant transformation of dysplastic lesions [ 9 ], there is increasing interest in identifying additional biomarkers that could increase diagnostic precision and prognostic accuracy. From this perspective, the CD1a marker, which indicates the presence of immature Langerhans cells with a high capacity for antigen uptake [ 8 ], can serve as an indicator for assessing immunosurveillance and dysplasia progression. A thorough examination of the association between this marker and dysplasia grade could enhance our understanding of immune mechanisms and help identify prognostic indicators.

Prior studies on this subject have reported inconsistent findings, for instance, Upadhyay *et al* . [ 6 ] observed a significant decrease in the number of CD1a-positive cells in higher grades of OED and SCC. In contrast, Pellicioli *et al* . [ 8 ] reported an increased number of CD1a-positive cells in higher grades of OED and SCC.

The present study aimed to investigate the correlation between CD1a expression and various clinical and pathological parameters, such as dysplasia grade, inflammation severity, patient gender, and lesion location in oral leukoplakia with epithelial dysplasia. 

## Materials and Method

### Study Design and Sample Selection

The present study was approved by the Ethics Committee of Shahid Beheshti University of Medical Sciences (IR.SBMU.DRC.REC.1402.113). The samples consisted of 60 formalin-fixed paraffin-embedded tissue specimens (27 females and 26 males; mean age: 54 years, ranging from 27 to 90 years) submitted to the Department of Oral and Maxillofacial Pathology, School of Dentistry, Shahid Beheshti University of Medical Sciences. These included 30 cases of mild, 16 of moderate, and 7 of severe epithelial dysplasia (all of which were clinically diagnosed as leukoplakia based on the three-tier WHO grading system in 2022), and 7 control samples (consisting of 5 irritation fibromas and 2 epulis fissurata). Of the 53 OED lesions, 25 (47%) were located in the tongue, 15 (28%) in the buccal mucosa, 4 (7%) in floor of the mouth, 3 (6%) in the labial mucosa, 3 (6%) in the hard palate and 3 (6%) in the gingiva. Hematoxylin and eosin (H&E) stained slides and paraffin blocks were evaluated by an experienced oral pathologist to verify the specimen adequacy. Demographic data, including patient age, gender, and lesion site were recorded.

### Inclusion and Exclusion Criteria

Eligible samples included those with a confirmed clinical diagnosis of leukoplakia and a histopathological diagnosis of epithelial hyperplasia with dysplasia. All of the dysplastic lesions were primary. Samples were excluded if they had inadequate tissue, poor fixation quality, or incomplete records.

### Immunohistochemistry

The staining procedure was performed using the Envision technique on 4-μm-thick sections. The tissue sections were deparaffinized and rehydrated through a descending ethanol series. For antigen retrieval, the slides were subjected to a 0.01 mM citrate buffer in a microwave oven for two intervals of 10 minutes each. A distilled water solution was utilized to block endogenous peroxidase activity for 10 minutes at room temperature. Following a rinse with phosphate-buffered saline (PBS), the primary antibody (ready to use rabbit monoclonal antibody, clone EP80, Master Diagnóstica, Spain) was applied. The sections were washed in PBS for 10 minutes and then incubated with a secondary antibody (Mouse/Rabbit PolyVue™ HRP/diaminobenzidine tetrahydrochloride (DAB)) for 30 minutes at 37°C. After another rinse with PBS, the colorimetric development was carried out using the 3,3’ DAB substrate, followed by counterstaining with hematoxylin. The expression of positive control consisted of the skin specimen. For negative control, the primary antibody was replaced by a nonimmune serum.

### Morphological Identification and Quantification

**Figure 1:**
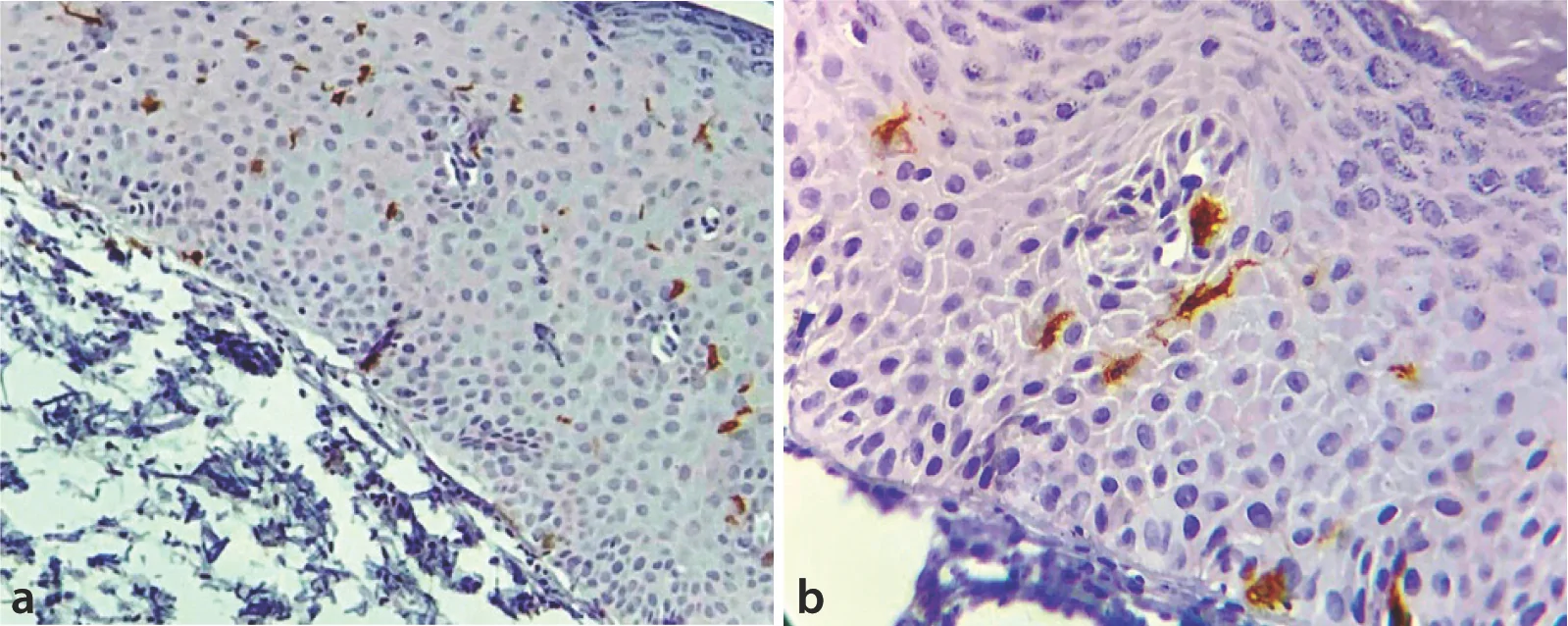
Non-dysplastic (control) samples. CD1a-positive cells (brown staining) with cytoplasmic projections (**a:** IHC100×, **b:** IHC400×)

**Figure 2:**
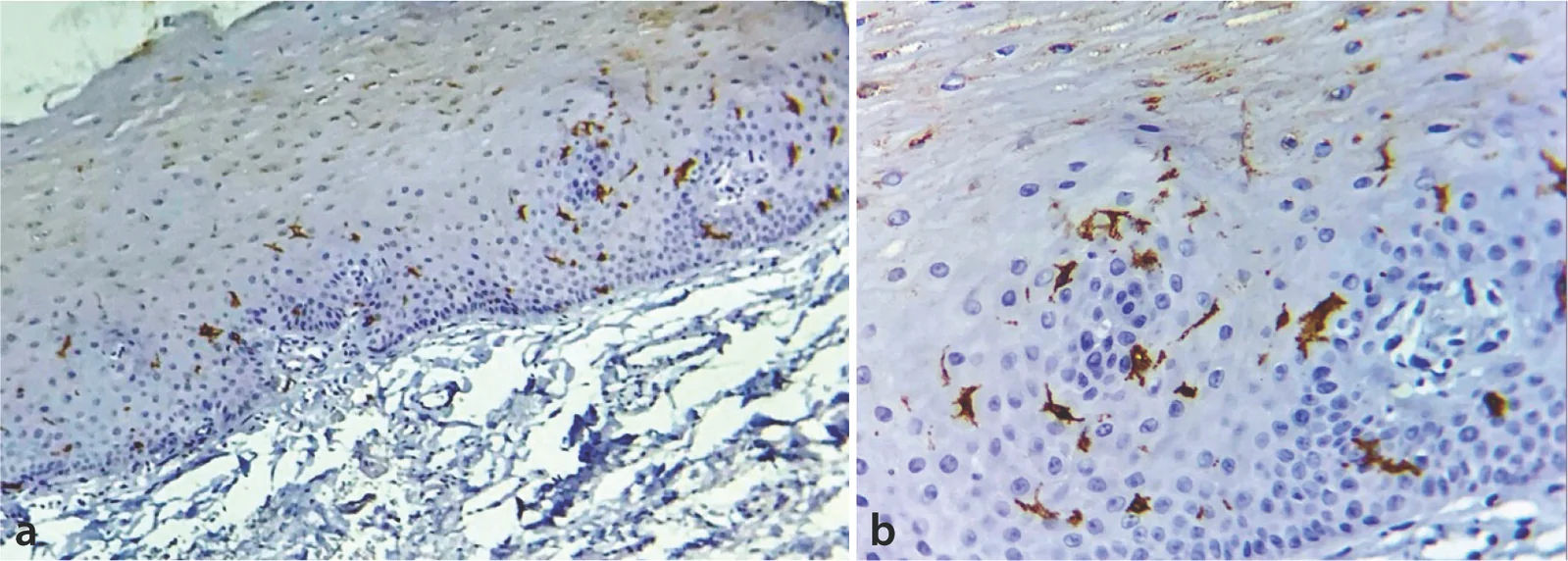
Mild dysplasia. CD1a-positive cells (brown staining) with cytoplasmic projections (**a:** IHC100×, **b:** IHC400×)

**Figure 3:**
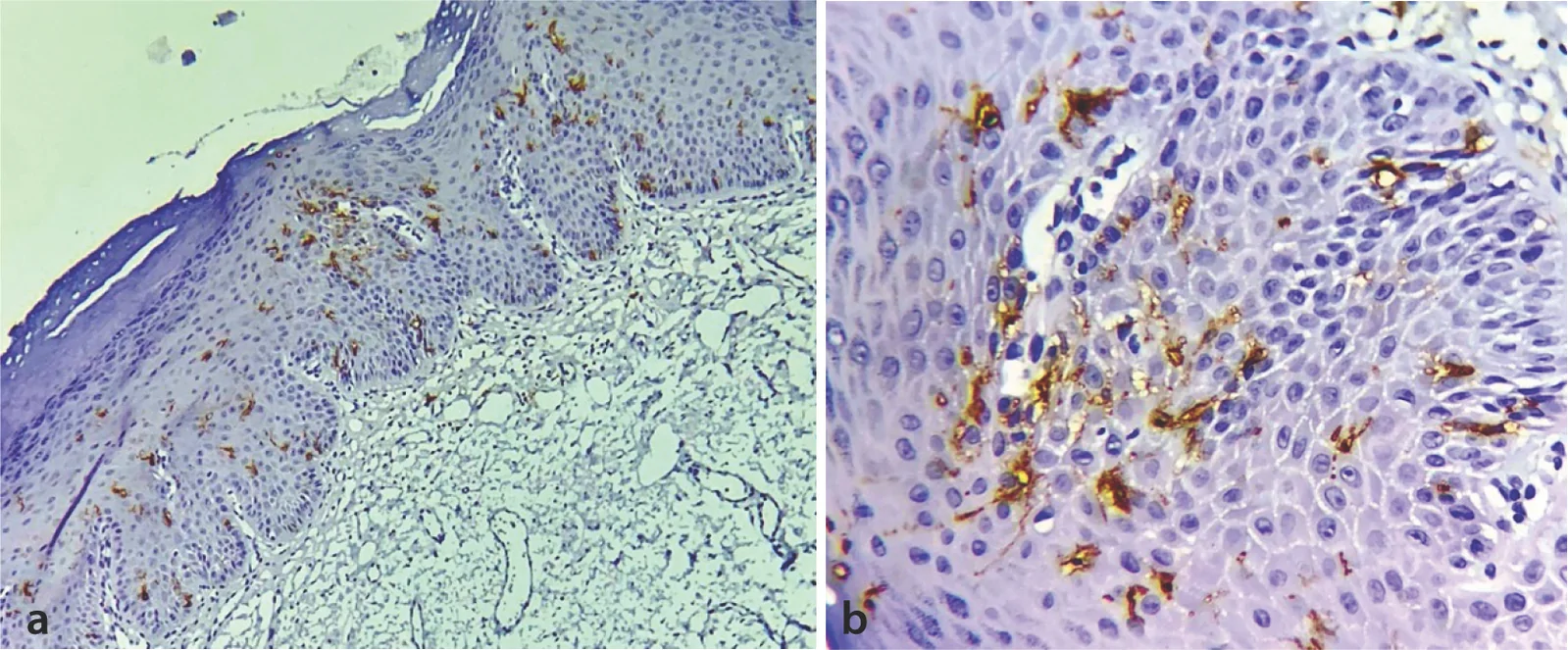
Moderate dysplasia. CD1a-positive cells (brown staining) with cytoplasmic projections (**a:** IHC100×, **b:** IHC400×)

**Figure 4:**
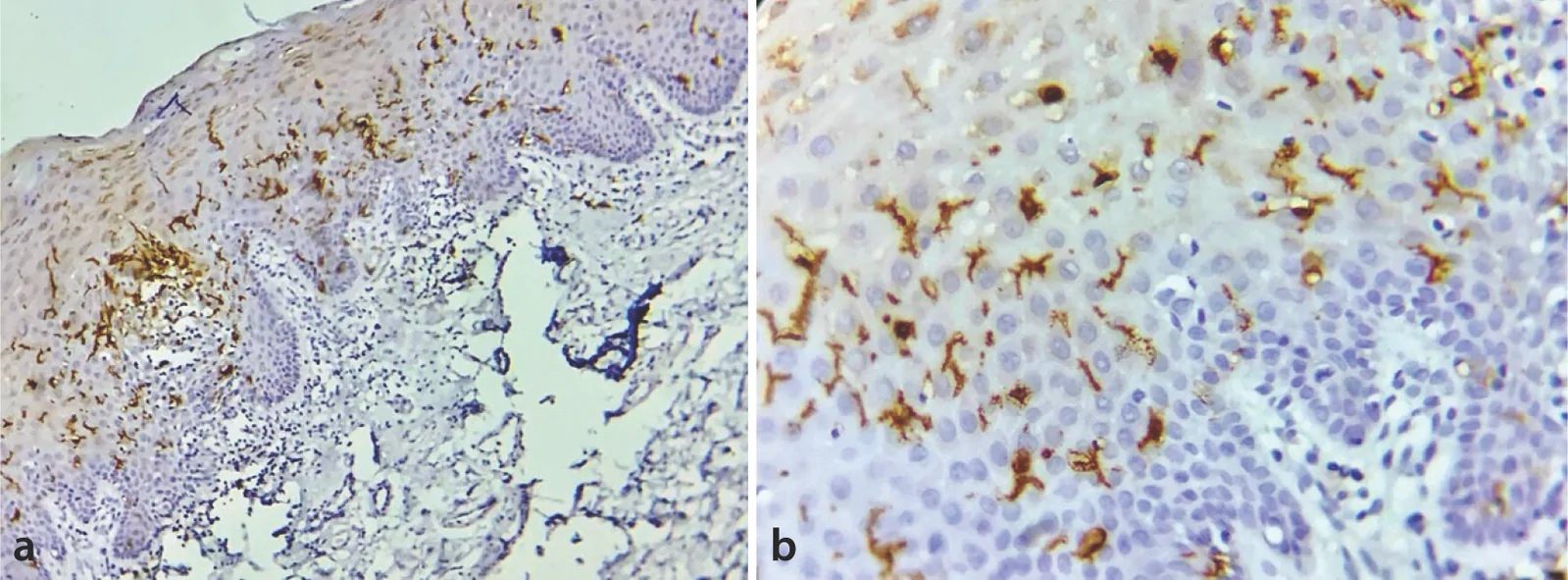
Severe dysplasia. CD1a-positive cells (brown staining) with cytoplasmic projections (**a:** IHC×100, **b:** IHC×400)

All slides were evaluated in a blinded fashion by an oral pathologist. CD1a-positive Langerhans cells were quantified following the method described by Pellicioli *et al* . [ 8 ]. Hotspot areas, defined as regions with the highest density of positive cells, were first identified under 10× magnifications. Subsequently, positively stained cells were counted in five high-power fields (40×), and the average count was calculated, as shown in Figures 1-4. Counting was performed throughout the whole epithelial layer as a total count. The cells were typified as Langerhans cells under the following criteria: (a) CD1a staining was found localized, which appeared brownish in color, (b) round/ovoid brown stained cell body, which must be visible completely, and (c) at least one dendritic process must be present from the cell surface [ 6 ]. 

### Classification of Dysplasia and Inflammation

Dysplastic samples were classified using both the WHO (2022) system (mild, moderate, severe) and the binary system (low risk, high risk) [ 3 ], providing a comprehensive assessment of dysplasia severity and its association with CD1a expression. Similarly, inflammation was graded into three categories (mild, moderate and severe) according to the method described by Farahani *et al* . [ 11 ]. The intensity of inflammatory cell infiltration was evaluated using the following semiquantitative scale: (0) absent, (1) mild (slight, scattered), (2) moderate (slightly diffuse or prominent scattered inflammatory cells), and (3) severe (prominent, diffuse).

### Statistical Analysis

Descriptive statistics were used to summarize the data. The Shapiro-Wilk test was applied to assess the normality of data distribution. Spearman’s correlation was used to evaluate associations between ranked and continuous variables. The Mann-Whitney U test was employed to compare CD1a expression between genders and between normal and dysplastic tissues due to non-parametric distribution. The Kruskal-Wallis test was used to compare CD1a expression across different locations. Statistical analyses were performed using SPSS version 28, with a *p* Value< 0.05 considered statistically significant. 

## Results

CD1a expression was higher in dysplastic tissues than in control samples (mean±SD: 9.77±6.17vs. 5.77±4.78), although this difference did not reach statistical significance (*p*= 0.071) (Table 1). In the binary grading system, CD1a expression was significantly higher in high-risk lesions compared to low-risk ones (*p*= 0.044) (Table 2), whereas in the WHO classification, the highest CD1a expression was observed in the moderate dysplasia group, however, this difference was not statistically significant (*p*= 0.069) (Table 3).

**Table 1:** Statistical analysis of CD1a expression between control and dysplastic tissues

Group	N	Mean±SD	Statistical Test	*p* Value
Control	7	5.77±4.78	Mann-Whitney U	0.071
Epithelial dysplasia	53	9.77±6.17		

**Table 2:** Statistical analysis of CD1a expression among different dysplasia grades based on the Binary classification

Dysplasia Severity (N)	Mean±SD of CD1a	Min-Max of CD1a	Spearman’s rho (r)	*p* Value
Control (7)	5.77±4.78	2.2–16.2	0.260*	0.044*
Low grade (30)	8.90±5.84	1.4–24.2		
High grade (23)	10.89±6.53	1.6–30.0		

**Table 3:** Statistical analysis of CD1a expression among different dysplasia grades based on the WHO classification

Dysplasia Severity (N)	Mean±SD of CD1a	Min-Max of CD1a	Spearman’s rho (r)	*p* Value
Control (7)	5.77±4.78	2.2–16.2	0.236	0.069
Mild (30)	8.90 ±5.84	1.4–24.2		
Moderate (16)	11.40±6.58	1.6–30.0		
Severe (7)	9.74±6.78	2.6–19.8		

While CD1a expression showed a positive correlation with inflammation severity, this association did not achieve statistical significance (*p*= 0.137) (Table 4). Nevertheless, a statistically significant association was found between the inflammation severity and dysplasia grade in both classification systems (*p*= 0.015 for WHO; *p*= 0.023 for binary).

**Table 4:** Statistical analysis of CD1a expression between different levels of inflammation severity

Inflammation Severity (N)	Mean±SD of CD1a	Min-Max of CD1a	Spearman’s rho (r)	*p* Value
Mild (31)	7.96±5.20	1.4–23.0	0.194	0.137
Moderate (14)	11.54±7.99	1.8–30.0		
Severe (15)	9.98±5.61	2.8–19.8		

No statistically significant correlation was detected between CD1a expression and patients’ age (*p*= 0.707), gender (*p*= 0.516), or lesion anatomical site (*p*= 0.951). Most lesions were located on the tongue (n=25), whereas the highest CD1a expression was seen in the hard palate. 

## Discussion

In this study, CD1a expression was higher in dysplastic tissues compared to normal oral epithelium; however, the difference was not statistically significant. This recruitment can be attributed to the uptake of new antigens produced by the dysplastic cells, although it is likely that additional regulatory factors may also influence its expression. 

Our findings are consistent with those of Kindt *et al* . [ 12 ] and Ohman *et al* . [ 7 ], both of which reported an increased presence of CD1a cells in dysplastic and malignant lesions compared to normal tissues. These findings support the hypothesis that tumor or dysplastic cells may actively recruit dendritic cells through chemokines such as MIP-3α, thereby contributing to enhanced immune surveillance. Kindt *et al* . [ 12 ] also reported that a higher density of CD1a-positive cells in tumor-involved lymph nodes was associated with a better prognosis. Likewise, Goldman *et al* . [ 13 ] observed that CD1a-positive cell density in peritumoral areas correlated with lower recurrence rates. Upadhyay *et al* . [ 6 ] fou-nd increased CD1a expression, particularly in the basal and supra-basal layers of dysplastic epithelium and in the superficial layers of OSCC. Souto *et al* . [ 14 ] also reported higher dendritic cell density in larger and severe dysplastic changes, further supporting our findings that immune activity increases with dysplasia severity.

Mechanisms proposed for this increased infiltration include mitotic activity of Langerhans cells [ 6 ], inhibition of their migration to lymph nodes, and chemokine driven recruitment to dysplastic regions. These changes may reflect the immune system’s effort to present tumor antigens and initiate a defensive response [ 12 ].

Nevertheless, conflicting results have been reported. For example, Pellicioli *et al* . [ 8 ] found no significant difference in CD1a expression between OSCC and OED, while Costa *et al* . [ 15 ] reported higher CD1a-positive cell counts in normal tissues compared to dysplastic or malignant lesions. Similarly, Da Silva *et al* . [ 10 ] reported significantly lower CD1a cells in both OSCC and leukoplakia cases compared to control groups. These discrepancies may stem from methodological differences or variations in sample characteristics.

In our analysis, higher CD1a expression was correlated with increasing epithelial dysplasia severity. Within the WHO classification system, CD1a levels were higher in moderate dysplasia compared to mild cases. Similarly, in the binary classification, CD1a expression was significantly higher in high-grade lesions compared to low-grade ones, suggesting a positive association between dysplasia grade and increased CD1a expression.

These results are supported by Kindt *et al* . [ 12 ], who reported a higher number of Langerhans cells in more severe grades of dysplasia, although they did not observe a similar pattern across different stages of OSCC. Pellicioli *et al* . [ 8 ] also observed a higher density of CD1a+ cells in high-grade OED than in low-grade cases, suggesting an immune response recognizing altered epithelial cells. This may reflect an increased presence of immature dendritic cells in poorly differentiated lesions actively engaged in presenting tumor-associated antigens to T lymphocytes.

In contrast, Upadhyay *et al* . [ 6 ] found a decrease in Langerhans cell numbers as dysplasia progressed. They hypothesized that severe dysplastic lesions may lack sufficient immune support due to reduced recruitment of Langerhans cells following prolonged exposure to inflammatory stimuli. This could weaken immune surveillance and facilitate malignant transformation. Also, they have seen that in mild-moderate dysplasia, the basal and suprabasal epithelial zones exhibited the highest density of CD1a cells.

One of our findings in this study was the significant positive correlation between the grade of dysplasia and the severity of inflammation. Since inflammatory infiltration includes immune cells such as T cells and dendritic cells [ 16 ], these findings support the potential role of inflammation in promoting CD1a expression and suggest that higher-grade dysplasia may contribute to a microenvironment characterized by increased infiltration of immune cells and higher immune activity, with more CD1a cells present in the tissue. This could be explained by the finding that CD1a cells express high levels of specific receptors (CCR1, CCR5, and CCL20) for the inflammatory chemokines CCL3, CCL5, and CCL20 [ 15 ]. Therefore, the accumulation of CD1a cells may reflect a response to these inflammatory chemokines, aiming to recognize and process antigens in the inflamed dysplastic epithelium.

The current study indicates that age and gender were not significantly associated with CD1a expression, as no statistical difference was observed between male and female patients or across different age groups. Similarly, although the highest level of CD1a expression was found in the hard palate, this difference was not statistically significant. These results suggest that CD1a expression is primarily influenced by the grade of dysplasia and inflammation severity rather than demographic or anatomical factors. While our findings showed no significant association between lesion site and CD1a expression, some studies have reported location-dependent immune variations, particularly in SCC of the vermilion of the lip, suggesting that immune responses may vary across anatomical regions due to local immunological differences [ 15 ].

The current study suggests that the presence of CD1a cells may reflect an active immune response during the progression of epithelial dysplasia. However, inconsistencies in the literature highlight the complexity of dendritic cell behavior in oral epithelial lesions and emphasize the need for further studies and larger sample sizes to elucidate these interactions. 

## Conclusion

Based on the present study, CD1a appears to play a role in the dysplastic transformation process in oral leukoplakia. The increased Langerhans cells in dysplastic tissues may represent an immune system response aimed at presenting tumor-associated antigens and stimulating an immune reaction. Furthermore, the association between higher grades of dysplasia and increased inflammation suggests that high-grade dysplasia may contribute to a microenvironment characterized by more significant inflammatory cell infiltration, enhanced immune activity, and increased presence of CD1a-positive Langerhans cells. 
